# Effect of Maternal Age on the Proliferation and Osteogenic Differentiation of Human Umbilical Cord Stem Cells

**DOI:** 10.1155/bmri/1430675

**Published:** 2026-10-13

**Authors:** Hana Dennaoui, Mira Abouchi, Adel Hussein, Rabih Roufayel, Ziad Fajloun, Chaza Harmouch

**Affiliations:** ^1^ Department of Biology Faculty of Sciences 3 Lebanese University Michel Slayman Tripoli Campus Ras Maska Lebanon ul.edu.lb; ^2^ Laboratory of Applied Biotechnology (LBA3B) Azm Center for Research in Biotechnology and its Applications EDST Lebanese University Tripoli Lebanon ul.edu.lb; ^3^ College of Engineering and Technology American University of the Middle East Kuwait aum.edu.kw

**Keywords:** human umbilical cord, maternal age, osteogenic differentiation, proliferation, stem cells

## Abstract

Human umbilical cord (UC), once regarded as medical waste, has emerged as a valuable and ethically acceptable source of mesenchymal stem cells (MSCs) due to its high availability and regenerative potential. Among UC‐derived tissues, Wharton′s jelly (WJ) is particularly rich in MSCs, making it a promising candidate for cell‐based therapies; however, factors influencing the biological properties of these cells, including maternal age, remain insufficiently understood. This study is aimed at evaluating the impact of maternal age on proliferation and differentiation of MSCs from the WJ of human UC. In this comparative study, UC from healthy mothers grouped into five age groups (25–45 years old) were collected and treated to assess their proliferation and differentiation in vitro. The results indicate that the rate of proliferation and osteogenic differentiation are inversely associated to maternal age. The maternal age effect is important for clinical applications based on the use of MSCs from WJ.

## 1. Introduction

The term “stem cell” appeared in 1883 [1]. A stem cell is a cell that retains its ability to proliferate throughout the life of an individual. After division, the daughter cells can remain as stem cells and self‐renew or they can differentiate. The characteristic “self‐renewal” trait makes stem cells a repair system for reconstructing cells in many tissues that have been lost as a result of injury or disease [2]. Stem cells vary according to their type; each type possesses a certain degree of plasticity where some stem cell exhibit higher degrees of plasticity when compared with others. Additionally, stem cells have a unique ability to protect their telomeres which maintains their length [3]. This contributes towards the self‐renewal capacity of stem cells.

Mesenchymal stem cells (MSCs) represent a heterogeneous population of multipotent, adult, and fibroblast‐like cells. They have attracted much attention during the last decade in the field of regenerative medicine, mainly because of their hematopoiesis‐supporting property, abundant production of soluble growth factors and cytokines, and their ability to differentiate to specific cell types [4]. The use of MSCs in cell therapy appears to be due to different mechanisms: transdifferentiation, cell fusion, stimulation of angiogenesis, secretion of cytokines or beneficial growth factors, stimulation of endogenous stem cells′ proliferation, inhibition of apoptosis, and immunomodulatory effects [5].

Wharton′s jelly (WJ), the gelatinous connective tissue inside the umbilical cord (UC), represents a promising and ethically favorable source of MSCs. WJ‐derived MSCs (WJ‐MSCs) are multipotent, capable of differentiating into cells of the mesodermal lineages (osteocytes, chondrocytes, and adipocytes), and, in some studies, showing markers suggestive of even wider differentiation potential. They are collected at birth via noninvasive methods, pose far fewer ethical dilemmas than embryonic stem cells, and exhibit immunomodulatory properties, high proliferation rates, and low immunogenicity [6].

Among the key characteristics of WJ‐MSCs are their adherence to plastic in culture, morphology resembling fibroblasts, expression of MSC surface markers (such as CD73, CD90, and CD105), and negative expression for hematopoietic and endothelial markers (such as CD45 and CD34) [7]. They also express, though to a lesser extent than embryonic stem cells, certain pluripotency‐associated genes like OCT4, SOX2, and NANOG [8], and some embryonic markers (such as SSEA‐4), though they do not form teratomas in vivo. Their differentiation potential is robust, including osteogenic differentiation [9], making them of special interest for bone repair and regenerative medicine.

However, the functional quality of WJ‐MSCs can vary. Several intrinsic and extrinsic factors may influence key parameters such as proliferative capacity, yield, and lineage‐specific differentiation (including osteogenic differentiation). The cell microenvironment (niche) influences the proliferation of WJ‐MSCs. The concentration of oxygen, a very important component of the cellular niche, plays a role in maintaining plasticity and proliferation of stem cells. It has been shown that hypoxia ([O_2_] ~2.5%) ameliorates proliferation of MSCs [10]. WJ‐MSCs extracted from the UC of mothers exhibiting gestational diabetes, a common metabolic disorder in pregnant women, showed lower proliferation rates when compared with cells extracted from healthy mothers [11]. Other factors such as expression of genetic factors, maternal health and diet, and, most importantly, maternal age also affect the functional quality of WJ‐MSCs [12]. Studies have shown maternal age negatively correlates with yield of WJ‐MSCs, marker expression (such as CD105 and CD29), and expression of pluripotency/stemness genes (such as SOX2 and POU5F1) in WJ‐MSCs [13]. A study assessing maternal and neonatal factors in 79 UC samples found that WJ‐MSC yield decreases with increasing maternal age, whereas neonatal birth weight and gestational age positively correlated with yield [14]. Another study showed that expression of CD105 and CD29 in WJ and the vascular components of the UC (umbilical artery and vein) declines with increasing maternal age [15]. Moreover, SOX2 expression in WJ‐MSCs was significantly higher in mothers aged 34 or younger versus older mothers. SOX2 (SRY‐box transcription Factor 2) is a key transcription factor involved in maintaining pluripotency and self‐renewal in embryonic and certain adult stem cells. It functions as part of a core regulatory network alongside other transcription factors, such as OCT4 (POU5F1) and NANOG, all of which are crucial for maintaining an undifferentiated state. In MSCs, including those derived from WJ, SOX2 expression is considered a marker of stemness and regenerative potential [13].

Maternal diet and metabolic status play a significant role in determining the quality of WJ‐MSCs. Poor metabolic conditions—such as obesity or nutrient imbalance—are associated with reduced cell yield, slower proliferation, altered or diminished osteogenic differentiation, and epigenetic modifications [16]. These factors can potentially compromise the utility of WJ‐MSCs in regenerative medicine. On the other hand, a balanced maternal diet, avoidance of nutrient deficiency (e.g., calcium, phosphate, and essential vitamins), and management of metabolic health presumably support higher quality WJ‐MSCs.

Finally, it has been shown that chitosan/hyaluronic acid (CS/HA) multilayer films serve as biocompatible substrates for the culture of WJ‐MSCs. The CS/HA films, developed using layer‐by‐layer assembly techniques, supported cell adhesion, proliferation, and viability, indicating that they provide a favorable microenvironment for stem cell growth. These findings suggest that CS/HA multilayer films are promising candidates for use in cell culture of WJ‐MSCs to evaluate their proliferation and osteogenic differentiation [17].

This article is aimed at examining in detail how maternal age modulates the proliferation and osteogenic differentiation potential of WJ‐MSCs seeded on CS/HA films. Understanding these influences is critical to standardizing the selection and application of WJ‐MSCs in regenerative therapies.

## 2. Materials and Methods

### 2.1. Primary Culture of WJ‐MSCs of Human UCs

Fresh human UC were obtained after full‐term births with informed consent using the guidelines approved by the hospitals in Tripoli, Lebanon. Briefly, after rinsing in Hank′s balanced salt solution (HBSS) (Sigma) solution, cords were aseptically stored at 4°C in sterile saline solution until processing. Next, UC vessels were removed manually from cord segments, and the exposed WJ was cut into very small pieces or explants of approximately 1–2 mm. These explants were cultured in proliferation medium: alpha‐MEM (Sigma) supplemented with 10% decomplemented FBS (Sigma) and 1% antibiotics (penicillin‐streptomycin and Amphotericin B) and incubated at 37°C in normoxic environment (21% of dioxygen [O_2_] and 5% of carbon dioxide [CO_2_]). The daily phase contrast microscopic examination reveals the adhesion, good spreading, and morphology of MSCs at the bottom of 6‐well plates and shows the formation of small cell colonies in fibroblast form (CFU‐F). After 6 days of culture, the pieces of the jelly are removed from the wells. The cells are left in culture until 80% confluence is obtained. Then, after cell counting, cells are seeded in flasks at 3000 cells/cm^2^, and medium is changed twice a week until the fourth passage.

### 2.2. Characterization of WJ‐MSCs

This technique is used to identify membrane proteins called differentiation clusters (DCs), whose presence or absence determines the phenotype of cells in proliferation or differentiation.

#### 2.2.1. Immunophenotyping by Flow Cytometer

To detect cell surface antigens, cultured adherent cells after detachment with Trypsin‐EDTA were washed with phosphate‐buffered saline (PBS) (Gibco). Cells were immunolabelled with the following antihuman antibodies: CD90‐FITC, CD34‐FITC, CD44‐FITC, and CD73‐PE (BD Pharmingen, France). As controls, mouse IgG1‐PE, IgG1‐FITC, and IgG1‐HLA‐DR (BD Pharmingen, France) were used. At least 10,000 events were acquired on BD FACSCalibur flow cytometer, and the results were analyzed using WinMDI v2.8 software.

#### 2.2.2. Proliferative Profile of WJ‐MSCs

Our study was carried out by comparing the proliferative capacity of MSCs from 25 cords belonging to 25 women of five age groups: Group 1 (20–25), Group 2 (26–30), Group 3 (31–35), Group 4 (36–40), and Group 5 (41–45). The evaluation of the proliferative capacity of the WJ‐MSCs for the different age groups was studied in P3 after 2 weeks of culture by various tests: (i) number and distribution of nuclei/cm^2^, (ii) cell proliferation by crystal violet staining, and (iii) fluorescence microscopy/marking of the cytoskeleton.

#### 2.2.3. Cell Morphology

WJ‐MSCs cultured were observed by inversed microscopy (Leica MC 170 HD) to evaluate their morphology after 3, 7, 15, and 21 days of culture.

##### 2.2.3.1. Evaluation of Cell Proliferation by Crystal Violet Staining

Crystal violet is a dye that interacts with intracellular components. Cells that undergo cell death lose their ability to adhere and are then lost in the cell population, reducing the amount of violet crystal staining in a culture. At D15, the P3 cells were incubated with crystal violet for 15 min. The Triton X‐100 (permeabilizing the cells) was used as a negative control. Dye extraction was done by washing with SDS, and the absorbance was measured at 570 nm. The amplitude of cell adhesion was quantified by measuring the optical density of the crystal violet eluted at 570 nm. Cells treated with triton and then marked with crystal violet were used as a negative control. The relative optical density of WJ‐MSCs minus the optical density of negative control was compared for the different age groups.

##### 2.2.3.2. Cytoskeleton Visualization

F‐actin cytoskeleton was labelled using Phalloidin coupled with Alexa 488 (Invitrogen, France). After 2 weeks of culture, WJ‐MSCs were fixed with 4% paraformaldehyde for 15 min, permeabilized with PBS/Triton X‐100 (0.1%) for 15 min and blocked with PBS/BSA (1%). Cells were then incubated for 45 min at 37°C with Phalloidin (1/100). After two washes with PBS, nuclei were counterstained using the dye DAPI (Calbiochem; VWR international), a DNA intercalating agent, for 5 min at room temperature. The labelled cells were mounted on slides to be observed by fluorescence microscopy (Zeiss microscopy, ×20 magnification) using the (485Ex/538Em) spectral line.

##### 2.2.3.3. Osteogenic Potential of WJ‐MSCs

###### 2.2.3.3.1. Induction of Osteogenic Differentiation

Osteoblastic differentiation was induced by a mixture of *α*MEM containing 10% FBS, 100 U/mL antibiotics, 10 mM sodium *β*‐glycerophosphate (Sigma), 100 nM dexamethasone (Sigma), and 0.25 mM of sodium ascorbate (Sigma). The culture medium was renewed every 3 days during a culture period of 15 days. After this culture period, the cells were fixed in 1x PBS containing 4% PAF for 10 min at 4°C, and then rinsed with PBS.

###### 2.2.3.3.2. Immunolabeling of Osteocalcin and Osteopontin

Immunolabeling targeted the main noncollagenic proteins constituting the organic part of the extracellular matrix of osseous tissue: osteopontin (early marker) and osteocalcin (late marker), and was carried out according to the following protocol: The cells were fixed with a 4% (w/v) PAF solution, blocked with 1% BSA, labelled with an antiosteocalcin antiosteopontin antibody (polyclonal antimouse IgG, Dako, France, 1/100). Negative controls were also used in each assay to suppress autofluorescence of the primary and/or secondary antibodies. In addition, the cells were labelled with DAPI in order to visualize their nuclei. The results were observed and photographed by fluorescence microscopy (Zeiss microscope). All experiments were performed in *n* = 3 independent biological replicates. For single cell analysis, a total of *n* = 20 cells were randomly selected and analyzed per experimental group.

###### 2.2.3.3.3. Marking With Alizarine Red

The revelation of osteogenic differentiation was also achieved by staining with alizarin red. It is used to identify calcium deposits during the process of osteogenic differentiation and thus the mineralization of the extracellular matrix. Calcium forms a red complex with alizarin red by chelation.

A 1% (v/v) aqueous solution of alizarin red adjusted with soda to pH 4.2 was added to each well over a period of 30 sec to 5 min depending on the appearance of red/orange coloration. The samples were then abundantly rinsed with PBS and then observed by fluorescence microscopy. Color‐thresholding analysis was used to quantify calcium mineralization.

### 2.3. Statistical Analysis

Quantitative data are expressed as the mean ± standard error of the mean (SEM) or mean ± standard deviation (SD), as indicated in the respective figure legends. For comparisons involving only two experimental groups, statistical significance was determined using an unpaired Student′s *t*‐test. For multiple group comparisons across the various age cohorts, a one‐way analysis of variance (ANOVA) was performed, followed by Tukey′s Honestly Significant Difference (HSD) post hoc test for pairwise differences. In all cases, a *p* value of less than 0.05 was considered statistically significant ( ^∗^ *p* < 0.05,  ^∗∗^ *p* < 0.01,  ^∗∗∗^ *p* < 0.001, and  ^∗∗∗∗^ *p* < 0.0001). All statistical analyses and graphical renderings were performed using GraphPad Prism (GraphPad Software, San Diego, California, United States) and online ANOVA calculator (Statistics Kingdom).

## 3. Results

### 3.1. Phenotypic Characterization of WJ‐MSCs by Flow Cytometry

The mesenchymal phenotype of UC WJ‐MSCs was determined by flow cytometry. P0 cells showed strong positive expression for the mesenchymal markers evaluated: CD73, CD90, and CD44, while showing negligible expression levels for hematopoietic markers (CD34 and CD45). A sequential gating strategy was used to identify the WJ‐MSC population and exclude debris and cell aggregates. Initially, cells were identified based on their forward scatter (FSC) and side scatter (SSC) characteristics, allowing the main cell population to be distinguished from cellular debris. Doublets and aggregated cells were subsequently excluded using FSC‐A versus FSC‐H parameters, and the resulting singlet population was used for further analysis. The expression of the mesenchymal markers CD44, CD73, and CD90 was then assessed within the selected cell population, whereas CD34 and CD45 were evaluated to confirm the absence of hematopoietic markers. Appropriate unstained and/or negative controls were used to establish the positive and negative fluorescence gates. The same gating strategy was consistently applied to all samples. Representative flow cytometry gating plots are provided in Figure 1.

**Figure 1:**
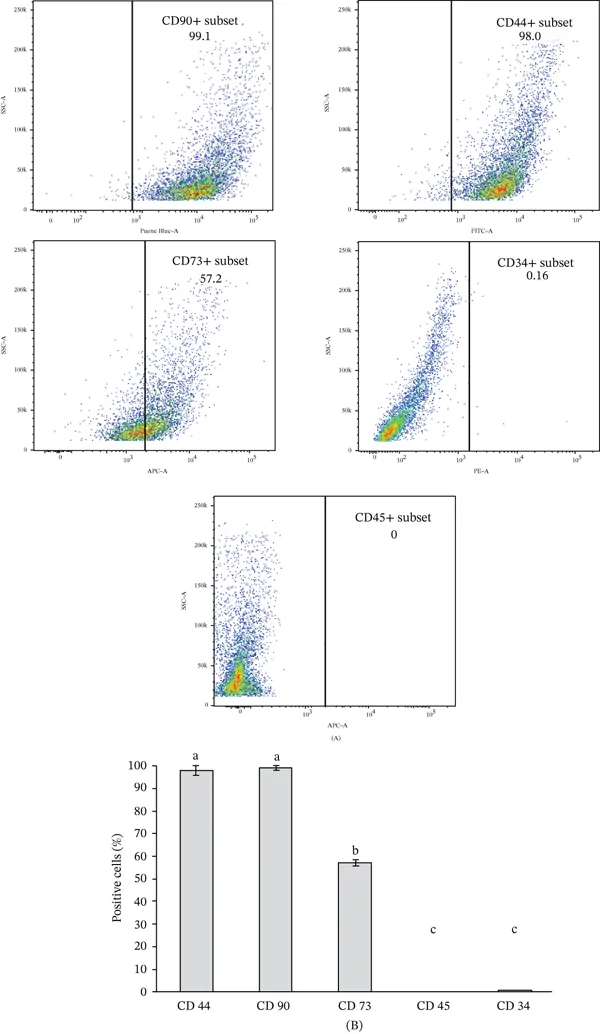
Cell characterization. (A) Phenotypic characterization of WJ‐MSCs from different age groups in P0 by flow cytometry. Analysis of phenotypic markers showed significant expression of mesenchymal markers (CD73, CD90, and CD44) and negligible expression of hematopoietic markers (CD34 and CD45). Data are presented as mean ± SD (*n* = 3). B) Statistical analysis was performed using a one‐way ANOVA (*F*(4, 20) = 5697.1, *p* < 0.001) followed by Tukey′s HSD post hoc test for multiple comparisons. Different lowercase letters above the bars denote statistically significant differences between groups (*p* < 0.05), whereas groups sharing a common letter are not significantly different (*p* > 0.05).

### 3.2. Comparison of the Proliferative Profile of WJ‐MSCs From Mothers of Different Age Groups

After phenotypic characterization of WJ‐MSCs acquired from 25 UCs obtained from mothers in five age groups: Group 1 (20–25), Group 2 (26–30), Group 3 (31–35), Group 4 (36–40), and Group 5 (41–45), with each age group represented by five different cords, the evaluation of the proliferative capacity of the WJ‐MSCs for the different age groups was carried out in P3 after 2 weeks of culture by different tests: (i) evaluation of MSC morphology by inverted phase contrast microscope, (ii) nucleus count and distribution/cm^2^, (iii) violet crystal cell proliferation, and (iv) fluorescence microscopy/cytoskeletal labelling. The women contributing to this study were healthy and signed a consent allowing us to use the cords for scientific research purposes.

#### 3.2.1. Evaluation of MSC Morphology by Inverted Phase Contrast Microscopy

The spread of the cells was examined daily during the P0 passage using the inverted phase contrast microscope (Leica MC 170 HD), and images were taken to assess the cell appearance and shape, as well as the confluence (Figure 2). After 3 weeks of culture, the cells in P0 were 100% confluent and a trypsinization step was done at this stage in order to pass into P1, P2, and P3. For the P1, P2, and P3 passages, the cells took less time to reach complete confluence.

**Figure 2:**
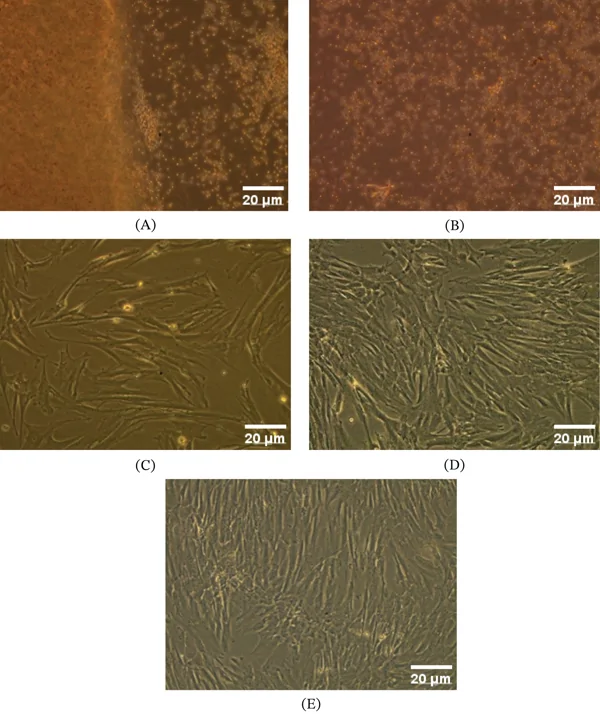
Cell morphology. Photos taken by inverted phase contrast microscope (Leica MC 170 HD) of MSCs extracted from UC in P0: (A) after 3 days of culture, (B) after 4 days of culture, (C) 1 week, (D) 2 weeks, and (E) 3 weeks. The cells appear confluent after 3 weeks of culture and retain their fibroblastic‐like morphology from D3 to D21 (objective ×20).

#### 3.2.2. Distribution of Cell Nuclei/cm^2^

After 2 weeks of culture, 140 nuclei were observed on the slide representing the first age group, 120 nuclei for the second age group, 100 for the third, and almost 50 for the last two age groups (Figure 3). These results show that the proliferation of WJ‐MSCs is inversely proportional to maternal age: the younger the mother, the higher the proliferation of MSCs.

**Figure 3:**
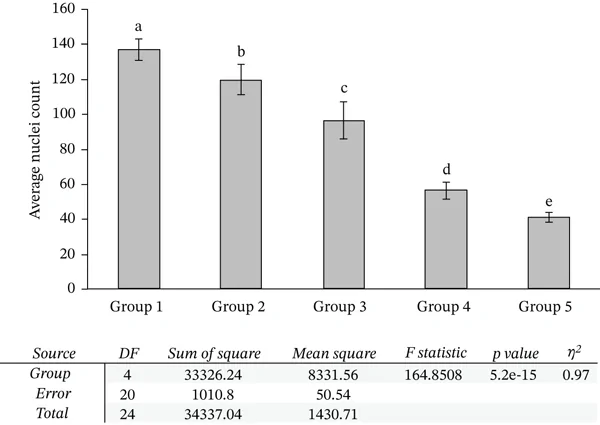
Cell count. The number of cell nuclei of adherent WJ‐MSCs on the glass at D15 for the different age groups studied was calculated by manual counting technique. Data are presented as mean ± SD. Statistical analysis was performed using a one‐way ANOVA (*F*(4, 20) = 164.85, *p* < 0.001) followed by Tukey′s HSD post hoc test for multiple comparisons. Different letters (a–e) indicate statistically significant differences between groups (*p* < 0.05).

#### 3.2.3. Evaluation of Cell Proliferation by Crystal Violet Staining

Cells treated with Triton and then crystal violet (Triton) were used as a negative control to compare cell proliferation among the different age groups studied. The optical density of Triton was set to 1 and the other densities were calculated relative to Triton. The results showed a decreasing optical density from Groups 1 to 5, which confirms the results of the cell count (Figure 4). Therefore, from the results of the count and crystal violet staining, we can confirm that the number of adherent cells is higher in the MSCs from the UCs of young women (20–35).

**Figure 4:**
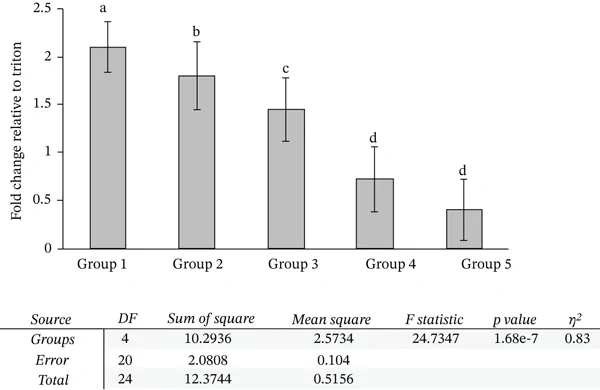
Cell adhesion. The adhesion of WJ‐MSCs of women of different age groups was determined by crystal violet staining after 15 days of culture on glass slides. Data are expressed as mean ± SD (*n* = 5 per group). Statistical significance was determined using a one‐way ANOVA (*F*(4, 20) = 24.73, *p* < 0.001) followed by Tukey′s HSD post hoc test for multiple comparisons. Different letters (a–d) above the bars indicate statistically significant differences between groups (*p* < 0.05), whereas groups sharing a letter (Groups 4 and 5) are not significantly different from one another.

#### 3.2.4. Evaluation of Cell Spreading by Labelling Actin Filaments With Phalloidin

Spreading of the actin cytoskeleton was evaluated via phalloidin labelling in order to assess the quality of adherent cells (Figure 5). Cells from the first age group have a fibroblastic‐like appearance (characteristic of MSCs) and appear well spread out in 2D (Figure 5A). The cells of the second (Figure 5B) and third age groups (Figure 5C) appear to be spread out as well, but less confluent than those of the preceding group. For Groups 4 (Figure 5D) and 5 (Figure 5E), cellular debris appears on the slides observed, indicating poor quality of the MSCs extracted from these cords. Thus, we can conclude that the quantity and quality of cells from women over 35 years of age remain poor compared with those who are younger. Evaluation of cell viability will be necessary to confirm all previous results.

**Figure 5:**
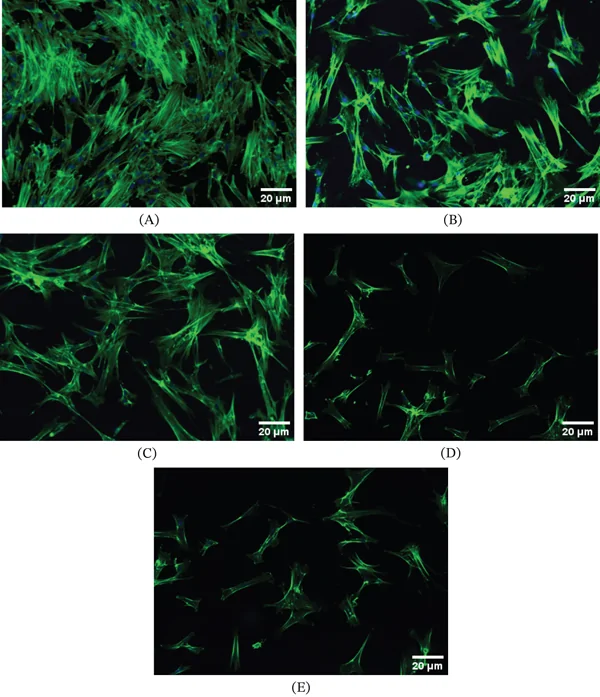
Cytoskeleton visualization. Photos of phalloidin‐labeled WJ‐MSCs after 2 weeks of culture on glass, actin (green) and nuclei (blue) filaments for the five age groups studied (Zeiss microscope, objective ×20).

### 3.3. Osteogenic Potential of WJ‐MSCs

The following figures were obtained after observation under a fluorescence microscope. The results show positive labelling of osteocalcin (Figure 6) and osteopontin (Figure 7) for MSCs from mothers aged 20–35 (A), whereas there is no labelling for MSCs from mothers aged 36–45 (B). The results of alizarin red staining (Figure 8) confirmed the osteogenic potential of MSCs from younger mothers (Figure 8A) compared with those of older mothers (Figure 8B). Our results show an influence of the mother′s age on the osteogenic differentiation potential of MSCs from WJ of the UC.

**Figure 6:**
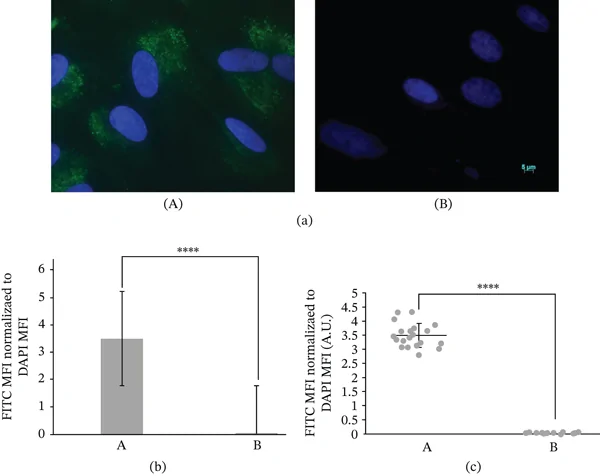
(a) Photographs of dual immunofluorescent (blue) and osteocalcin (FITC) labeling of MSCs from mothers of (A) age group 20–35 and those from the (B) age group 36–45 (Zeiss objective microscope  ^∗^oil *n* = 3). (b) Histogram showing the osteocalcin mean fluorescence intensity (MFI). (c) FITC MFI (representing osteocalcin) was normalized to DAPI MFI to acquire osteocalcin MFI in fold change. (A) Age groups 20–35. (B) Age groups (35–45). Results are ± SEM, *n* = 3 (independent biological replicates). Scatter plot showing individual cell osteocalcin MFI between age groups: (A) Age groups 20–35 and (B) age groups 35–45. Results are ± SEM. *n* = 20 (randomly selected cells) ( ^∗∗∗∗^ *p* < 0.00001).

**Figure 7:**
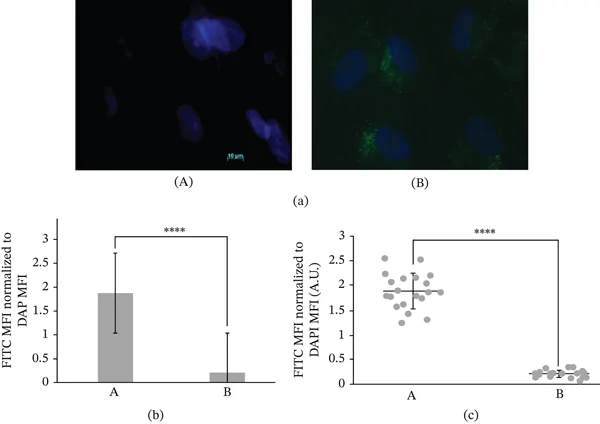
(a) Photographs of dual immunofluorescent (blue) and osteopontin (FITC) labeling of MSCs from mothers of (A) age groups 20–35 and those from the (B) age groups 36–45 (Zeiss objective microscope  ^∗^oil *n* = 3). (b) Histogram showing the osteopontin mean fluorescence intensity (MFI). (c) FITC MFI (representing osteopontin) was normalized to DAPI MFI to acquire osteopontin MFI in fold change. (A) Age groups 20–35. (B) Age groups 35–45. Results are ± SEM, *n* = 3 (independent biological replicates). Scatter plot showing individual cell osteopontin MFI between age groups: (A) Age groups 20–35 and (B) age groups 35–45. Results are ± SEM. *n* = 20 (randomly selected cells) ( ^∗∗∗∗^ *p* < 0.00001).

**Figure 8:**
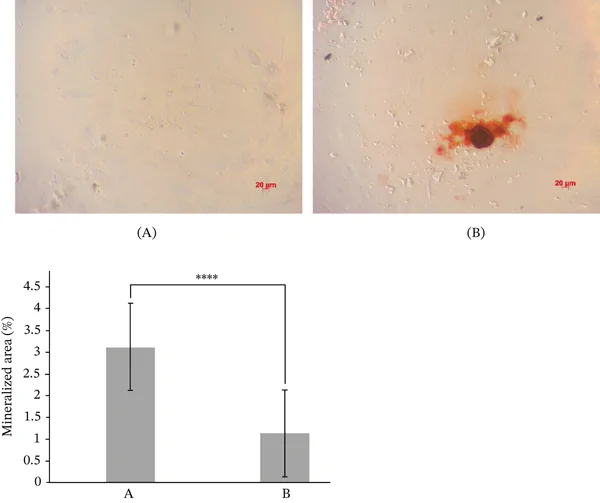
At the top: Evaluation of deposits of calcium mineralization after 15 days of osteogenic differentiation (culture in monolayer), after staining by the alizarine red of MSCs from mothers of (A) age groups 20–35 and those of the (B) age groups 36–45 (Zeiss objective microscope  ^∗^oil *n* = 3). At the bottom: A histogram showing the quantification of alizarin red staining using color‐thresholding analysis. Mineralization is expressed as the percentage of the total surface area. (A) Age group 20–35 years. (B) Age group 36–45 years. Results are expressed as mean ± SEM (*n* = 3;  ^∗∗∗∗^ *p* < 0.00001).

## 4. Discussion

The effect of aging on the regenerative capacity of living organisms and its impact on biological activities has been the subject of numerous previous studies, but few studies conducted to assess the effect of age on fetal stem cells and more particularly stem cells of the UC. These cells have many advantages such as the simple and noninvasive extraction techniques, their high proliferative capacity, and their accelerated differentiation time [18].

Numerous factors have been shown to influence MSC proliferation over the past several years. These include the composition of the culture medium (including growth factors and serum content), cell seeding density, the characteristics of the culture surface, the oxygen fraction [19], and mechanical stresses [20]. Factors associated with the maternal donor can influence both the proliferative capacity and osteogenic potential of MSCs. The mother′s age, health status, and pregnancy complications have all been shown to impact the quality of MSCs derived from the UC and placenta. For example, studies have found that conditions like gestational diabetes and preeclampsia have been linked to changes in the differentiation potential and growth of neonatal MSCs [21].

To evaluate how maternal age influences the proliferative and differentiating capacity of MSCs, we isolated MSCs from the WJ of human UC from healthy donors. These cells were sorted into five distinct maternal age groups, from youngest to oldest, for comparative analysis. We established a series of tests to specifically assess the proliferative capacity of these MSCs. Before testing, all isolated cells were confirmed to meet the basic criteria for MSCs, including a fibroblastic‐like morphology, plastic adherence, and the expression of key mesenchymal markers (CD73, CD90, and CD44).

Our tests indicate that the proliferative capacity of WJ‐MSCs is negatively correlated with maternal age. Results showed a marked decrease in cell proliferation, as evidenced by a lower nuclei count and reduced crystal violet staining in cells from older donors compared with younger ones. Furthermore, microscopic analysis revealed differences in cellular structure. While younger donors′ cells displayed a robust arrangement of actin filaments, cells from donors aged over 35 years exhibited initial signs of cellular debris formation, despite retaining a fibroblastic‐like morphology. The accumulation of this debris was notably higher in samples from donors over 40 years of age.

Our results confirm the data of Huang et al. who suggested that the young donor′s UC represents a relatively more efficient source of MSCs [21]. They speculated that the UC cells of older donors show a reduced proliferation capacity. Thus, age has a more significant impact on the proliferative power of MSCs; this can be attributed to a diminished functional state of the organs of older mothers. These organs, having an important role in the support of MSCs, offer the suitable microenvironment allowing the good development of these cells [22].

Our results demonstrated that WJ‐MSCs obtained from young donors exhibit significantly higher osteogenic differentiation potential compared with those derived from aged donors. Specifically, WJ‐MSCs from the young group undergo more rapid osteogenic differentiation, as evidenced by an accelerated rate of matrix mineralization and enhanced deposition of calcium nodules during in vitro assays. This increased osteogenic capacity in younger cells is likely associated with their superior proliferative activity and higher metabolic rate. To our knowledge, no studies discovering the effect of maternal age on the differentiation potential of WJ‐MSCs have been reported. However, a study conducted by Mahmoud et al. [13] showed that maternal age correlated negatively with stemness marker expression which suggests that older donors may have cells with lower differentiation potentials. Consequently, targeting WJ‐MSCs from young donors represents a more promising strategy for bone tissue engineering applications, as these cells can promote more efficient bone matrix formation and ensure robust extracellular matrix production necessary for effective bone regeneration [23].

## 5. Conclusion

In summary, our findings demonstrate that maternal age exerts a significant influence on the biological characteristics of Wharton′s jelly‐derived Mesenchymal Stem Cells (WJ‐MSCs). Cells isolated from younger donors display enhanced proliferative activity, better structural integrity, and superior osteogenic potential compared with those obtained from older donors. The decline in proliferation and differentiation capacities observed with increasing maternal age suggests that age‐related physiological changes in the maternal environment may alter the niche that supports fetal stem cell development. These results underscore the importance of considering donor age as a key variable in both experimental design and clinical applications involving perinatal stem cells. Consequently, WJ‐MSCs from young donors appear to represent a more suitable and reliable source for regenerative medicine and bone tissue engineering, owing to their high proliferative capacity, efficient osteogenic differentiation, and robust extracellular matrix production. Through this work, we were able to open up new avenues in the field of tissue engineering that suggest that maternal age is one factor that determines donor selection for WJ‐MSC therapies. Future studies should further elucidate the molecular mechanisms underlying this age‐dependent decline to optimize the therapeutic use of WJ‐MSCs across different donor populations.

## Author Contributions

Conceptualization: C.H. Data curation: H.D., M.A., and A.H. Formal analysis: H.D., R.R., and C.H. Visualization: Z.F. Methodology: H.D., M.A., and A.H. Project administration: C.H. Writing—original draft: H.D. Writing—review and editing: C.H.

## Funding

No funding was received for this manuscript.

## Disclosure

All authors read and approved the final version of the manuscript.

## Ethics Statement

This study was approved by the Institutional Review Board (IRB) of the University of Balamand (Approval Number: IRB‐REC/o/025‐28/0125; Approval Date: January 29, 2025).

## Conflicts of Interest

The authors declare no conflicts of interest.

## Data Availability

The data that support the findings of this study are available from the corresponding author upon reasonable request.
